# Developing a Pharmacist-Centered Novel Antimicrobial Stewardship (AMS) Approach for Healthcare in Pakistan: A Grounded Theory Study

**DOI:** 10.3390/antibiotics14121235

**Published:** 2025-12-08

**Authors:** Parniya Akbar Ali, Shaheer Ellahi Khan, Abdul Momin Rizwan Ahmad

**Affiliations:** 1 Department of Public Health, Health Services Academy, Islamabad 44000, Pakistan; shaheer@hsa.edu.pk; 2 Association for Social Development, Islamabad 44000, Pakistan; 3 Institute of Health Policy, Management and Evaluation, Dalla Lana School of Public Health, University of Toronto, Toronto, ON M5T 3M7, Canada; 4 Department of Human Nutrition and Dietetics, NUST School of Health Sciences, National University of Sciences & Technology (NUST), Sector H-12, Islamabad 44000, Pakistan; 5 Department of Health Sciences, University of York, York YO10 5DD, UK

**Keywords:** antimicrobial resistance, antimicrobial stewardship, pharmacists, barriers, facilitators, one health, Pakistan

## Abstract

Background: Antimicrobial resistance (AMR) is one of the most significant global health threats of the 21st century, endangering healthcare systems worldwide as a silent pandemic. Despite the globally recognized role of pharmacists as antimicrobial stewards, their involvement remains limited in antimicrobial stewardship (AMS) endeavors in Pakistan. Methods: By utilizing the Straussian grounded theory methodology, this study aimed to develop a pharmacist-centered novel AMS approach for healthcare in Pakistan in order to enhance the engagement of pharmacists in AMS and reduce the burden of AMR in Pakistan. Through 13 semi-structured in-depth interviews with pharmacists and AMS experts, this study explored the facilitators and obstacles faced by pharmacists in Pakistan pertaining to their participation in AMS. Results: The findings highlighted the underutilization of pharmacists in AMS owing to persistent policy, institutional, and resource-level barriers. Several facilitators were also identified, including institutional ownership and pharmacist-prescriber-patient (3P) communication. The evidence generated informed the development of the pharmacist-centered novel AMS approach, which recommends extending AMS policy support to pharmacists, improving One Health interdisciplinary collaborations, promoting pharmacist-led behavior change campaigns, granting prescribing rights to pharmacists for minor ailments, and advancing AMS trainings and education. Conclusions: Formally integrating pharmacists into AMS efforts is the need of the hour to contain the consequences of AMR in Pakistan.

## 1. Introduction

The discovery of antimicrobial medications considerably altered the course of treatment and containment of infectious diseases. Not only have antimicrobials conferred substantial benefits on contemporary medicine and health, they have also saved countless lives by providing adequate protection against numerous harmful and potentially fatal pathogenic microorganisms [1,2]. However, the emergence of antimicrobial resistance (AMR) has profoundly undermined this progress, and it is endangering the healthcare systems worldwide as a silent pandemic [2,3]. AMR is listed among the top ten most substantial global health threats of the 21st century, with an estimated 5 million people dying from it each year, a number significantly greater than the mortality rates from HIV/AIDS and malaria combined [4,5].

The consequences of AMR are severe in low- and middle-income countries (LMICs), and Pakistan is not immune to its detrimental effects, as well [6]. In 2019, around 1.27 million deaths resulted from drug-resistant infections in Pakistan, with 1 in 5 of these deaths occurring in children under 5 [7]. Some of the major obstacles critically affecting the future of antibiotics in Pakistan are the presence of unauthorized medical professionals, self-medication, and unrestricted access to antibiotics [8]. Such challenging times urgently demand stringent action in Pakistan, particularly in the realm of antimicrobial stewardship (AMS), which constitutes all the activities, programs, and interventions that intend to optimize the appropriate use of antimicrobials [9]. Although Pakistan has exerted considerable efforts to curb AMR, including the adoption of the AMR National Action Plan (AMR-NAP) by the utilization of the One Health approach, it continues to be burdened by significantly high AMR prevalence rates [10,11]. Hence, the findings bring to light the ineffectiveness of the current AMS initiatives and warrant the imperative need of alternative measures, including focused and concise AMS efforts, in order to effectively take on the threat of AMR in Pakistan.

Pharmacists, with their thorough knowledge in appropriate medication use, along with patient safety and counseling, are uniquely positioned to play a crucial role in AMS initiatives [12]. Worldwide, pharmacists are acknowledged for undertaking a number of interventions to promote AMS in various healthcare settings, such as the formulation of evidence-based guidelines, the provision of education and training initiatives, along with the review and audit of antimicrobial regimens and outcomes [13]. The role of pharmacists in AMS is well documented, and even in their traditional dispensing capacity, pharmacists have undertaken a number of strategic measures to address AMR [14]. A research study performed in the UK revealed the effectiveness of pharmacist-led education-based AMS initiatives in controlling AMR [15]. In addition, a review published in 2019 in the U.S. reported a significant decrease in antibiotic prescribing and an improvement in prescribers’ adherence to guidelines after involving pharmacists in AMS efforts [16]. The U.S. Centers for Disease Control and Prevention (CDC) recommends appointing pharmacists in either a leadership or co-leadership position for the effective implementation of any AMS program and highlights their crucial role in developing and implementing AMS-related policies [17]. Furthermore, pharmacists are recognized for contributing significantly as antimicrobial stewards by the American Society of Health-Systems Pharmacists (ASHP) [18].

In Pakistan, the majority of the pharmacies are operated and managed by laypeople, owing to a country-wide shortage of qualified and registered pharmacists, and this mismanagement is further exacerbating the threat of AMR [19]. The literature highlights the blatant lack of interest in the promotion of the pharmacy profession in Pakistan and the absence of vacancies in pharmacist-related organizations as a major flaw in the Pakistani healthcare system, which is potentially fueling the irrational consumption of antibiotics [20]. In addition, studies conducted in Pakistan have revealed that the pharmacists demand a comprehensive multidisciplinary AMS approach, which is pharmacist-led and could function as the cornerstone of successful AMS programs [8,21].

Despite a growing emphasis on AMS in Pakistan, a significant gap remains surrounding the crucial role of pharmacists in leading AMS efforts. Prior research describes challenges but does not offer an approach that thoroughly explains how pharmacist-led stewardship could function in practice, particularly in resource-constrained environments. Hence, this study aimed to develop a pharmacist-centered novel AMS approach for healthcare in Pakistan, in order to expand the contribution of pharmacists in AMS programs and initiatives by exploring the facilitators and obstacles faced by pharmacists pertaining to their involvement in AMS in Pakistan. This research endeavor gathered the perspectives of not just the pharmacists but also the AMS experts in Pakistan to holistically reach conclusions. The generated evidence contributed to the development of the approach, which has the potential to strengthen the formal integration of pharmacists into the AMR response in Pakistan, as pharmacist-directed AMS efforts could just be the key to addressing the pressing challenge of AMR [22].

## 2. Results

A total of 13 semi-structured in-depth interviews were conducted. The characteristics of the study respondents, with their current role and practice setting (sector and facility type), along with the numbers assigned to the participants for anonymization and the mode of interview, are given in Table 1. Overall, two themes and twenty-three sub-themes were identified. The key themes were (1) structural, professional, and policy obstacles to pharmacist-centered AMS and (2) professional and systemic facilitators to pharmacists’ involvement in AMS. Theme 1 reflects the multiple constraints that limit pharmacists’ engagement in AMS, while theme 2 highlights the various factors that have the potential to facilitate the participation of pharmacists in AMS in Pakistan. These themes provide an integrated and thorough understanding of the obstacles and facilitators shaping pharmacist-centered AMS in Pakistan.

### 2.1. Theme 1—Structural, Professional, and Policy Obstacles to Pharmacist-Centered AMS

Several critical obstacles to the involvement of pharmacists in AMS in Pakistan were highlighted by the respondents.

#### 2.1.1. Absence of National AMS Policies and Programs

Respondents reported an absence of national AMS policies and programs as a barrier to the involvement of pharmacists in AMS.

“The biggest barrier is that we don’t have a national policy the current application of which is promoting pharmacists’ involvement in AMS.”

(R1)

A lack of AMS programs was also reported as an obstacle, with one participant commenting the following:

“Lots of hospitals have it in their policy that they don’t have such programs. Like we don’t have any AMS programs in our hospital.”

(R10)

#### 2.1.2. Lack of Antibiotic Dispensing Guidelines

An absence of antibiotic dispensing guidelines was highlighted by a number of participants, with some revealing their personal experiences surrounding dispensing without appropriate guidelines and protocols.

“In our setting, proper guidelines are not generated. The concept we have is that the doctor has written it, we [pharmacists] have to give it to the patient immediately.”

(R10)

#### 2.1.3. Absence of Administrative Ownership

Participants mentioned administrative barriers, including an absence of administrative ownership, as a hurdle in the way of pharmacist-centered AMS.

“The biggest barrier is that we [pharmacists] don’t have that place where if we want to do something, we can implement it. And the AMS programs, they can be more clearly done in such a place where pharmacists can be owned.”

(R3)

Pharmacists are often seen as dispensers by the administration at any workplace. A participant remarked,

“A big barrier is your administration, how much it is promoting you and promoting your suggestions. I mean usually it happens that the poor pharmacist is considered a mere dispenser in a pharmacy. It becomes difficult to change their mentality, that we are part of a team.”

(R9)

#### 2.1.4. Prescriber Dominant System

They study participants provided their views on the healthcare system of Pakistan and described it as prescriber dominant.

“There is only one formula in the local market which is that the doctor is the king in Pakistan.”

(R1)

Additionally, the hostile attitude of the prescribers toward pharmacists was also described by a number of respondents.

“In pharmacists’ daily round of wards, they get this hinderance that the medical specialists there don’t give them the liberty to interfere in their prescriptions.”

(R13)

#### 2.1.5. Lack of Prescriber-Pharmacist Communication

A lack of communication and interaction between the prescriber and the pharmacist was reported by the respondents as an obstacle.

“I would say that there is no interaction between pharmacists and doctors at all. We are in a separate field according to them and they are in a separate field according to us…”

(R8)

#### 2.1.6. Lack of Pharmacists’ Involvement in AMS

Participants reflected on their personal experiences and mentioned their own lack of involvement in AMS in their professional roles.

“I have never been a part of any AMS initiative in all the years I have worked as a pharmacist.”

(R9)

#### 2.1.7. Stakeholders’ Hesitance

Respondents reported the hesitation of stakeholders in involving pharmacists in AMS as a significant challenge.

“It could be that in our healthcare system, stakeholders are threatened by us that we will take their position, so they hesitate to include us in AMS.”

(R4)

#### 2.1.8. Absence of Qualified Pharmacists

An absence of pharmacists, especially in the community pharmacy setting, was mentioned by a number of participants as a barrier.

“In our society, the concept of pharmacies is very less, but the concept of medical stores is there. And there we don’t have pharmacists…”

(R6)

#### 2.1.9. Insufficient AMS Trainings, Workshops, and Education

Respondents highlighted insufficient AMS trainings, workshops, and education as an obstacle to the involvement of pharmacists in AMS. A participant commented,

“The first basic thing is our own education, our own trainings, we have not had such trainings, neither in our education, nor in our course book we had such things…”

(R4)

#### 2.1.10. Patients’ Personal Behaviors

Patients’ personal behaviors, including self-medication and a lack of compliance, were reported by the participants.

“The people of Pakistan, even if they cough a little, they will run for the antibiotic. They are addicted…”

(R9)

Another participant remarked,

“First of all, if there’s compliance, only then the people will complete the course. Usually what happens is that people take a dose once, they get relief and then they discontinue the antibiotic.”

(R8)

#### 2.1.11. Absence of Diagnostic Stewardship

Participants showed concerns regarding the absence of diagnostic stewardship and mentioned it as a barrier.

“There are a lot of challenges in diagnostics, including accuracy of diagnosing infections. Whatever the diagnostic tools are, culture tests etc., they are very lacking. There is no concept of cultures here…”

(R11)

### 2.2. Theme 2—Professional and Systemic Facilitators to Pharmacists’ Involvement in AMS

Respondents highlighted a number of factors that have the potential to enhance the involvement of pharmacists in AMS.

#### 2.2.1. AMS Policy and Governance Support

Respondents recommended strengthening AMS policies and governance to address the roles of both pharmacists and physicians.

“If there is such governance and policies that in community setup pharmacists never dispense antibiotics without prescription and the physicians restrict antibiotic prescribing in prescriptions…”

(R3)

Some highlighted the need to formulate policies surrounding AMR and AMS. A respondent remarked,

“It is very important to make a comprehensive AMS policy.”

(R13)

#### 2.2.2. Interdisciplinary Collaborations

The promotion of interdisciplinary collaborations was discussed by a number of study participants, with some advocating for collaborations based on the One Health approach, in order to enhance the role of pharmacists in AMS.

“…You must be aware of One Health, we are actively involved in this and this should be promoted further…”

(R12)

#### 2.2.3. Institutional Support and Ownership

Participants highlighted extending institutional support to pharmacists to strengthen their role in AMS.

“When pharmacist will be owned in his domain by institutions, then the physician will know, that yes, in my setup the pharmacist matters…”

(R3)

#### 2.2.4. Prescribing Rights and Authority

Respondents cited examples from the practices of pharmacists around the world and recommended granting prescribing rights and authority to them for minor ailments to facilitate their involvement in AMS.

“Globally in multiple countries what’s happening now is that pharmacists have complete dominance in prescription practices and they can generally prescribe for minor conditions.”

(R1)

#### 2.2.5. Pharmacist-Prescriber-Patient (3P) Communication

Enhanced interaction between pharmacists and physicians with respect to the patients was reported as a potential facilitator.

“If we get to interact more with the patients during when doctors are treating the patients, like when a doctor is sitting to examine the patients, if a pharmacist is also made to sit there, it can be a huge facilitator…”

(R8)

#### 2.2.6. Emphasis on Prescribing Indicators

Respondents discussed the importance of prescribing indicators and recommended utilizing them while prescribing medicines.

“According to prescribing indicator we should fill prescriptions, which in Pakistan is very rare.”

(R3)

#### 2.2.7. Adherence to AWaRe (Access, Watch, Reserve) Framework

Another recommendation put forth by the participants was adhering to WHO’s AWaRe framework in prescriptions.

“…Pharmacists and physicians should give importance to the AWaRe classes and try to mention the Reserve class of antibiotics as little as possible.”

(R13)

#### 2.2.8. AMS Trainings and Workshops

Participants highlighted the promotion of AMS trainings and workshops in order to enhance the role of pharmacists in AMS.

“I think that wherever pharmacists are employed…those companies or hospitals should have trainings or campaigns in such a way that pharmacists should be trained at the company level.”

(R1)

#### 2.2.9. AMS Education and Certifications

Improving the knowledge of pharmacists on AMS through proper education and certifications was discussed by the respondents.

“I think that when we [pharmacists] are being trained, which starts from university, at the same time we should also be taught about AMS in a proper way…”

(R7)

#### 2.2.10. Pharmacist-Led Behavior Change Campaigns

Respondents recommended pharmacist-led behavior change campaigns in order to address the behavior of the prescribers toward pharmacists.

“We need to change the behavior through awareness. We need to bring behavioral change in our doctors and other healthcare providers, so that they are able to accept the role of pharmacists…”

(R12)

#### 2.2.11. Inclusion of Pharmacists in Ward Rounds

The inclusion of pharmacists in ward rounds was seen as a facilitator to promote pharmacists’ involvement in AMS.

“…Pharmacists should have clinical rounds every week, of every ward, medical ward, surgical ward…”

(R13)

#### 2.2.12. Prescription Audit and Feedback

Respondents recommended prescription audit and feedback to advance the involvement of pharmacists in AMS efforts.

“Pharmacists, especially clinical pharmacists, should do audit and feedback of prescriptions.”

(R13)

### 2.3. The Logic Model as a Framework for the Pharmacist-Centered Novel AMS Approach

**Figure 1:**
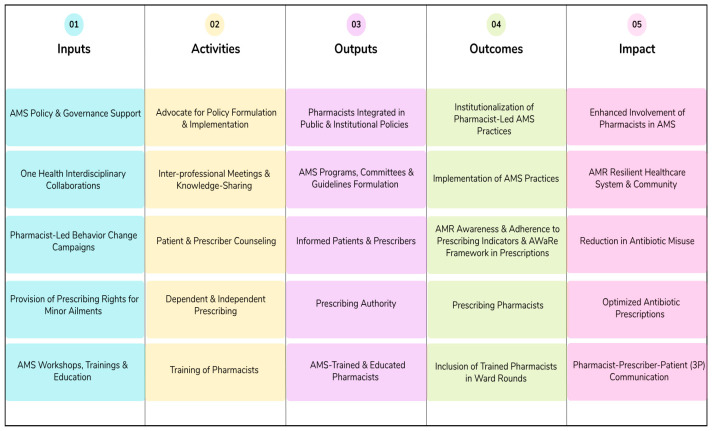
The pharmacist-centered novel AMS approach for healthcare in Pakistan.

The logic model, which is a visual representation that explains how a proposed strategy is an effective solution to the problem at hand and serves as a blueprint for change, was utilized as a guiding framework to develop the pharmacist-centered novel AMS approach from the above-mentioned results [23]. The results were organized under the five main components of the model, including inputs (resources, capacities, and enabling conditions necessary to implement something), activities (actions or interventions to conduct), outputs (direct products of the activities performed), outcomes (short-term or medium-term changes in response to outputs), and impact (broader, long-term effects), and the final approach was formulated (Figure 1). The sub-themes were integrated throughout the approach, with AMS policy and governance support; One Health interdisciplinary collaborations; pharmacist-led behavior change campaigns; provision of prescribing rights for minor ailments; and AMS workshops, trainings, and education as inputs. These five inputs were used to inform the activities, outputs, outcomes, and impacts, and collectively, they constituted the pharmacist-centered novel AMS approach for healthcare in Pakistan.

## 3. Discussion

This study aimed to develop a pharmacist-centered novel AMS approach for healthcare in Pakistan, in order to enhance the involvement of pharmacists in AMS programs and initiatives by exploring the facilitators and obstacles faced by pharmacists pertaining to their involvement in AMS. The developed approach (Figure 1) attempts to scale up the role of pharmacists in AMS in a systematic way, through tangible resources and intangible enablers. Overall, the approach recommends extending AMS policy support to pharmacists, improving One Health interdisciplinary collaborations, promoting pharmacist-led behavior change campaigns, granting prescribing rights to pharmacists for minor ailments, and advancing AMS trainings and education.

Health policy and governance efforts form the basis of any initiative as they are crucial to inform choices about the development of novel health programs [24]. Similarly, the developed approach highlights that in order to enhance the role of pharmacists in AMS, policy and governance support is essential, which requires policy advocacy and stakeholders’ engagement and acceptance of the role of pharmacists in Pakistan’s healthcare system. As duly underscored by some of the participants of the study, an absence of national AMS policies and programs that integrate the pharmacy profession and the hesitance of stakeholders regarding the acceptance of pharmacists in healthcare are unfortunately some of the most significant obstacles to the involvement of pharmacists in AMS. Considering these barriers, a study conducted in the UK called attention to acknowledging and involving pharmacists from various settings in the NAPs, in order to better leverage their untapped potential in AMS [25]. In addition, pharmacist-centered stewardship efforts have been shown to significantly reduce AMR by improving prescribing practices, reducing irrational antibiotic use, optimizing antibiotic therapy, and enhancing the clinical outcome of patients [26]. Hence, pharmacists must be formally integrated into public and institutional policies to enhance their role in AMS, so that pharmacist-led AMS efforts are institutionalized via the top-down approach as it is imperative to involve Pakistani pharmacists in the development and implementation of AMS initiatives at the community and hospital levels [27].

The fight against AMR requires an all-hands-on-deck strategy, and effective interdisciplinary collaborations are the need of the hour. A Canadian study on AMS described a lack of stakeholder collaboration and leadership as some noteworthy AMS barriers and identified the pressing need of communication and collaboration at all levels of healthcare [28]. Moreover, if there are any endeavors performed, pharmacists are often underutilized in such collaborations [29]. Interdisciplinary initiatives have been shown to improve infection prevention and control (IPC), enhance patient education on antibiotic consumption, and reduce inappropriate antibiotic use by up to 30% [30,31]. There is a dire need to promote a collaborative environment and involve all stakeholders to facilitate knowledge sharing and good antibiotic use practices in Pakistan. When asked about the facilitators to pharmacists’ involvement in AMS, the participants of this study recommended interdisciplinary collaborations, with some underscoring the significance of the One Health approach to holistically improve AMR in the country. In addition, pharmacists, in collaboration with other healthcare professionals, must develop, implement, monitor, and revise antibiotic dispensing and prescribing protocols and guidelines [32]. Therefore, One Health interdisciplinary collaborations must be promoted as only through effective partnerships, mutual understandings, and trust among the stakeholders involved can the patient care and outcomes be improved and resistance rates be lowered.

Behavior change is an integral part of any AMS program in order to ensure rational and optimal antibiotic use and combat AMR. There is an urgent need to facilitate the behavior change of not only the patients but also the healthcare professionals regarding unnecessary antibiotic use and prescribing, respectively. Pharmacists are the champions of medication counseling and education and play a crucial role in awareness-raising campaigns. Pharmaceutical counseling has been shown to improve medication adherence and compliance in patients, as well as enhance clinical outcomes and improve patient safety [33]. The personal behaviors of the patients are a significant challenge in AMS, with the practice of self-medication, pre-mature therapy discontinuation, and inconsistent use and misuse of antibiotics rapidly exacerbating the resistance patterns of pathogens and worsening the spread of AMR in Pakistan. In the UK, the pharmacists play a crucial role in raising awareness surrounding AMR and supporting AMS endeavors [13]. Additionally, pharmacist-led educational interventions are some of the most utilized interventions to improve judicious antibiotic use in communities [34]. Pharmacists in Pakistan must hold dedicated counseling sessions and workshops to educate doctors on the importance of adhering to prescribing indicators and the AWaRe framework of antibiotics in prescriptions. Similarly, diagnostic stewardship, which is the effective utilization of diagnostic tests to improve antimicrobial use, must be promoted in Pakistan, and antibiotics must not be prescribed without bacteria culture tests [35]. Pharmacists, with an expertise in educational interventions, must be empowered to counsel patients on their personal behaviors, as well as the prescribers on their prescribing habits and patterns. Such efforts collectively will not only raise awareness surrounding AMR in the country but also pave the way for pharmacist-led AMS efforts and interventions.

The approach also advocates for the provision of prescribing rights to pharmacists for minor ailments as a means to improve and optimize antibiotic prescribing. Many countries around the world have imparted prescribing authority to pharmacists, including Australia, Canada, the U.S., and the UK,^,^ with varying levels of authority in line with prescribing models [36]. In dependent prescribing, pharmacists have the authority to select, monitor, modify, or discontinue the pharmacotherapy, while independent prescribing involves patient evaluation, treatment initiation, and clinical outcomes management, all of which are performed by the pharmacist, with Canada and the UK being two of the most experienced developed countries utilizing the practice of prescribing by pharmacists for minor conditions [37,38]. In Pakistan, articles over the last decade or so have been advocating for the provision of prescribing rights to pharmacists for minor ailments [39]. Regarding the benefits of prescribing by pharmacists, a systematic review demonstrated positive results in the management of acute and chronic diseases by prescribing pharmacists, while another highlighted efficient division of workload between pharmacists and doctors and enhanced pharmacotherapy in the management of chronic conditions as some of the prospective benefits [40,41]. Owing to this, the approach puts forth the recommendation of granting prescribing rights and privileges to pharmacists in Pakistan, especially for acute and minor ailments, with the ultimate aim of optimizing antibiotics in prescriptions and reducing AMR rates in the country.

Insufficient AMS trainings, workshops, and education were emphasized by a number of study participants as a potential obstacle to pharmacists’ involvement in AMS. Pharmacists should be adequately equipped with the relevant skills and knowledge in order to carry out AMS activities effectively. Findings from a study performed to understand the role of pharmacists in AMS in Malaysia brought to light the absence of structured trainings as a significant hurdle in the way of pharmacist-led AMS interventions [42]. In the U.S., however, pharmacists undergo trainings in AMS through post-graduate residency programs, along with board certifications, and receive specialized trainings in infectious diseases to play their part as antimicrobial stewards [43]. Unfortunately, in LMICs, the capacity of pharmacists remains low owing to a scarcity of such initiatives [44]. In addition, the current study highlights that trained pharmacists must be included in ward rounds as in Pakistan, their crucial role in patient care in wards is not recognized, and they remain confined to mere dispensing even in hospital settings and continue to face exclusions. Babu and colleagues highlighted a number of benefits of including pharmacists in ward rounds, including reduced adverse drug reactions (ADRs), as well as improved and enhanced appropriateness of medicines [45]. Thus, the approach stresses upon addressing the knowledge gaps in AMR and AMS education through extensive trainings and advocates for the inclusion of trained clinical pharmacists in ward rounds across all hospitals of Pakistan. This has the potential to enhance pharmacist-prescriber-patient (3P) communication and optimize antibiotic use as multidisciplinary ward rounds have proven to reduce irrational antibiotic use [46].

This study is the first to promote the involvement of pharmacists in AMS by developing a pharmacist-centered novel AMS approach for healthcare in Pakistan, but its implementation must consider both short- and long-term feasibility. In the short term, strategies such as structured AMS trainings for pharmacists, patient and prescriber counseling on AMR, and improved documentation can be introduced within the existing healthcare settings. Long-term adoption will require systemic changes, including regulatory enforcement of pharmacist presence, expansion of the pharmacist workforce, and promotion of interdisciplinary AMS collaborations. The findings from the study are expected to aid policymakers and practitioners in formally integrating pharmacists in AMS efforts. However, this study also has numerous limitations that should be considered. An important limitation was the fact that the study explored the perceptions and viewpoints of mostly the pharmacists, with only two doctors as AMS experts. Future research should consider incorporating perspectives from other healthcare professionals, including nurses, pharmacy staff, and managers, as well as a large number of AMS experts, in order to provide a more comprehensive understanding of pharmacists’ involvement in AMS. Furthermore, this study had the presence of more hospital pharmacists than community pharmacists as in Pakistan, most community pharmacies are being run by non-pharmacists; therefore, generalizing the findings to the community pharmacy setting may be limited. Finally, there is a need to pilot this approach in Pakistan to evaluate its operational practicality and understand its true potential in enhancing the role of pharmacists in AMS.

## 4. Materials and Methods

### 4.1. Study Design

This study utilized the grounded theory methodology to qualitatively explore the obstacles and facilitators faced by pharmacists regarding their involvement in AMS in Pakistan. One of the major strengths of adopting such a methodology is that it provides a systematic and rigorous set of procedures and techniques of collecting, analyzing, and interpreting data in order to develop a theory or approach [47]. A Straussian grounded theory methodology was adopted, involving open and axial coding with memo writing [48]. In addition, a pragmatist research paradigm and epistemology (knowledge is always based on experiences) was employed because the goal was to develop a solution-oriented, substantive approach [49].

### 4.2. Study Participants and Sampling

Based on the principle of theoretical sampling and the belief that sample diversity can lead to a richer understanding of the phenomenon being explored, this study comprised a total of 13 participants, with 11 pharmacists and 2 AMS experts [48]. The study population, including AMS experts, as well as pharmacists, was purposively selected from across Pakistan. Purposive sampling is a non-probability sampling technique that involves the intentional selection of study participants with experiences and knowledge relevant to the over-arching research question [50]. In order to understand diverse perspectives on the obstacles and facilitators influencing pharmacists’ involvement in AMS and ensure that the developed approach did not overlook the perspectives of participants working across different sectors, pharmacists from various sectors with varying health infrastructures and with a basic understanding of AMS, as well as AMS experts directly involved in AMS initiatives, were selected. The literature does not make any numerical recommendations regarding the determination of sample size for a grounded theory study; hence, there was no predetermined sample size, and the decision to discontinue recruiting participants was based on the criterion of theoretical saturation [47]. Following this criterion, the participants were recruited and interviewed until the themes were well developed and the new data failed to generate information that added to the approach being developed. Saturation was achieved within the first 13 interviews.

### 4.3. Data Collection Technique and Tool

Semi-structured, in-depth interviews were conducted with pharmacists and AMS experts after obtaining informed consent. An interview guide with a set of interview questions and probes was developed by the first author (a pharmacist familiar with qualitative research) and the second author (a qualitative research expert) in order to give a structure to the interviews (Supplementary File S1). The interview questions were developed by incorporating the constructs of the Consolidated Framework for Sustainability Constructs in Healthcare, which were modified to address the obstacles and facilitators influencing pharmacists’ involvement in AMS [51]. The questions covered key topics, including the facilitators and obstacles surrounding the engagement of pharmacists in AMS. Initially, the interview guide also had a category that conceptualized the participants’ AMS awareness, which was iteratively updated after the first three interviews when new categories emerged. For example, the original question “What do you know about AMS?” was revised to “What roles and responsibilities do you think pharmacists should have in AMS?” to prompt more specific, experience-based responses. Overall, 13 interviews were conducted, and 10 interviews were taken face to face at the participants’ place of choosing. As 3 of the participants were based in other cities, their interviews were conducted online on Zoom, and the duration of each interview ranged from 15 to 20 mins. The respondents were first contacted and informed of the study, and interviews were conducted after taking informed consents from all the participants. The interviews were audio-recorded to ensure data capture and transcribed for analysis. As the data collection and analysis occurred concurrently, the ongoing review of the transcripts by the first author supported methodological rigor consistent with the principle of theoretical sampling [48]. All the interviews were conducted by the first author to ensure consistency, and field notes were recorded throughout the data collection process in order to refine prompts and probes.

The interview guide was pretested on 2 non-participating pharmacists from a private pharmacy in Rawalpindi. Resultantly, a few questions that yielded irrelevant responses were omitted from the guide, and some questions were simplified to elicit more meaningful responses. The test interviews were not analyzed for this study.

### 4.4. Data Analysis

Data analysis commenced after the first interview, continued throughout and after data collection, and involved constant comparison. The transcripts and the notes made about the concepts discussed in the interviews were constantly reviewed, which assisted in guiding the questioning in subsequent interviews. The aim of the analysis was to identify, develop, and relate the concepts that would form the building blocks of the approach [47]. In order to do so, an open and axial coding approach was adopted [48]. Initially, each recorded interview was transcribed verbatim in English, followed by the thorough reading and re-reading of the transcripts to develop familiarity with the data. Open coding involved line-by-line coding of the transcripts in order to develop provisional codes (i.e., descriptive words or short phrases). No preconceived codes were used in order to ensure the codes reflected the perspectives of the pharmacists and AMS experts and that the emerging approach was grounded in the data. Codes were continuously compared across transcripts to explore any similarities and irregularities in the participants’ experiences and, in some instances, revised based on new interview data.

Through axial coding, the codes were examined, any relationships between them were noted, and overlapping codes were grouped together to form broader themes and sub-themes. The themes and sub-themes were created when a large portion of the data would highlight a single concept. Lastly, in order to provide a clear structure to the approach, the logic model was utilized to visually represent the pharmacist-centered novel AMS approach that resulted from the themes and sub-themes. An example of how the raw data was transformed through initial coding, the development of the sub-themes, and the integration into the logic model is provided in Supplementary File S2. Memos and Microsoft Excel served as critical tools throughout the data analysis process.

### 4.5. Methodological Rigor

In order to enhance the methodological rigor of the study, various strategies underpinning the Straussian methodology were adopted [48]. As per this methodology, a theory is only considered valid if saturation is achieved. As previously stated, theoretical saturation was realized as new data did not yield more information, and the themes and sub-themes were sufficiently developed. Furthermore, the interview questions were kept open-ended, which allowed the pharmacists and AMS experts to provide detailed responses and share their experiences in their own words. In addition, theoretical sampling was utilized, and the research followed an iterative process of data collection and analysis. Moreover, the data was subjected to constant comparison, and memos were written to document reflections and refine emerging ideas about the data. Finally, interview quotes were embedded within the results to give the participants a voice and thoroughly support the themes and the analysis.

### 4.6. Reflexivity

The authors spent a substantial amount of time critically reflecting on their positions. They acknowledge that their backgrounds and previous knowledge of AMS had the possibility of impacting their interpretations of the data. Hence, reflexive field notes were kept, and regular debriefing sessions were conducted after the interviews, which ensured that the interpretations remained grounded in participants’ perspectives and minimized bias.

### 4.7. Ethical Considerations

This study was approved by the Institutional Review Board of Health Services Academy (protocol code 000832/HSA/MSPH-2023). The interviews were conducted by the first author, who was independent of the participants’ workplaces and had no relationship with them. All the participants provided informed consent. The informed consent form included the study’s purpose, its procedure, the voluntary nature of participation, as well as the potential risks and benefits to the participants. The interviews were audio-recorded and transcribed with the permission of the participants. In order to maintain anonymity, numbers were assigned to participants, and any identifying information was excluded from the transcripts. Throughout the study, all the data was stored on password-protected devices, only accessible to the researchers.

## 5. Conclusions

With the increasing burden of AMR in Pakistan and the growing need for AMS efforts, it is imperative to utilize the expertise of pharmacists and enhance their involvement as AMS stewards. This study not only addresses the underutilization of pharmacists in AMS endeavors in Pakistan but also formulates a novel pharmacist-centered AMS approach, by exploring the obstacles and facilitators influencing the involvement of pharmacists in AMS.

Drawing on participants’ viewpoints and suggestions, the approach recommends extending AMS policy support to pharmacists, improving One Health interdisciplinary collaborations, promoting pharmacist-led behavior change campaigns, granting prescribing rights to pharmacists for minor ailments, and advancing AMS trainings and education. There exists a necessity to implement this approach in Pakistan in order to comprehend its true potential in enhancing the role of pharmacists in AMS. Additional research is needed to validate and support the practicality of these findings. Furthermore, stringent AMS guidelines and protocols need to be formulated and implemented, and active sensitization of the stakeholders is required to acknowledge and encourage the expertise of pharmacists in Pakistan. Knowledge gaps in AMR and AMS education must be addressed through extensive trainings, and trained pharmacists must be included in ward rounds to reinforce the practice of clinical pharmacy in Pakistan. Formally integrating pharmacists into AMS efforts is the need of the hour, and comprehensive AMS programs and strategies must be introduced and adopted across all healthcare settings in Pakistan in order to bridge practice gaps, improve patient outcomes, and ease the overwhelming burden of AMR in Pakistan.

## Figures and Tables

**Table 1:** The characteristics of the respondents, including their assigned number, current role, practice setting and mode of interview.

S. No.	Respondent Numbers	Current Role	Sector and Facility Type	Mode of Interview
1.	R1	Clinical pharmacist	Private, clinic	Face to face
2.	R2	Hospital pharmacist	Private, hospital	Face to face
3.	R3	Retail pharmacist	Private, retail	Online
4.	R4	Hospital pharmacist	Private, hospital	Face to face
5.	R5	Industrial pharmacist	Private, industry	Face to face
6.	R6	Assistant manager, pharmacy services	Public, hospital	Online
7.	R7	Hospital pharmacist	Private, hospital	Face to face
8.	R8	Retail pharmacist	Public, retail	Face to face
9.	R9	Evening hospital pharmacist	Private, hospital	Face to face
10.	R10	Purchase pharmacist	Private, hospital	Face to face
11.	R11	Pharmacy manager	Private, hospital	Face to face
12.	R12	Senior scientist and team lead, AMR program	Public, research organization	Face to face
13.	R13	Chairman, AMS committee	Public, hospital	Online

## Data Availability

The raw data supporting the conclusions of this article will be made available by the authors on request.

## Supplementary Materials

The following supporting information can be downloaded at https://www.mdpi.com/article/10.3390/antibiotics14121235/s1. Supplementary File S1: Interview guide, Supplementary File S2: Example of the analytic progression from raw data to codes, subthemes and the logic model.

## Abbreviations

The following abbreviations are used in this manuscript:

ADRs	Adverse drug reactions
AMR	Antimicrobial resistance
AMS	Antimicrobial stewardship
ASHP	American Society of Health-Systems Pharmacists
AWaRe	Access, Watch, Reserve
CDC	Centers for Disease Control and Prevention
IPC	Infection prevention and control
LMICs	Low- and middle-income countries
NAP	National Action Plan
UK	United Kingdom
U.S.	United States
WHO	World Health Organization
