# Supplementary material for: Developing a Pharmacist-Centered Novel Antimicrobial Stewardship (AMS) Approach for Healthcare in Pakistan: A Grounded Theory Study
Source: Antibiotics (Basel). 2025 Dec 8;14(12):1235. doi: 10.3390/antibiotics14121235 (PMC12729923; doi:10.3390/antibiotics14121235)
Supplement: Supplementary file 1 [file antibiotics-14-01235-s001.zip › Supplementary File 1.pdf]

Supplementary File 1.

Interview Guide

The Type of Study and Semi-Structured Interviews

This study is expected to contribute to the development of a tailored, pharmacist-centered novel AMS approach for healthcare in Pakistan. There are no right or wrong answers to the questions I ask you. I want to know about the challenges and facilitators you think are important regarding the involvement of pharmacists in AMS initiatives in Pakistan. The interviews questions will be semi-structured and open-ended. Interview time will be around 15-20 minutes.

Introductory Question(s)

This section will focus on getting to know the interviewee, his/her background and the contextual environment in which they work/how it is related to AMS.

Questions Related to Obstacles to Pharmacists' Involvement in AMS
Framework Constructs: Organizational Setting, Resources, The People Involved

- What are the obstacles that limit a pharmacist's involvement in AMS?
  Probe: Can you provide specific examples?
- How do you perceive a pharmacist's interactions with doctors and other healthcare professionals regarding antibiotic use?
  Probe: Are these interactions collaborative or hierarchical?

Questions Related to Facilitators to Pharmacists' Involvement in AMS
Framework Constructs: Resources, The People Involved

- What would help a pharmacist contribute more effectively to AMS?
  Probe: What resources, tools, or training or would workshops or online courses be helpful?
- What support or changes would you/a pharmacist like from hospital management, policymakers, or other stakeholders to enhance your role in AMS?
  Probe: Are there policy gaps that need to be addressed?
- How can pharmacists collaborate more effectively with other healthcare professionals on AMS?
  Probe: Would regular multidisciplinary meetings help?

Questions Related to Designing a Pharmacist-Centered AMS Approach
Framework Constructs: Initiative Design and Delivery, Negotiating Initiative Processes

- In your opinion, what should a pharmacist-led AMS approach look like in Pakistan?
  Probe: Should it be more focused on education, regulation, or collaboration?
- What roles and responsibilities do you think pharmacists should have in such an approach?
  Probe: Should pharmacists be involved in direct patient education or prescriber audits?
- How can this approach be implemented effectively in hospital and community settings?
  Probe: What steps do you think would ensure its sustainability?
