# Supplementary material for: Developing a Pharmacist-Centered Novel Antimicrobial Stewardship (AMS) Approach for Healthcare in Pakistan: A Grounded Theory Study
Source: Antibiotics (Basel). 2025 Dec 8;14(12):1235. doi: 10.3390/antibiotics14121235 (PMC12729923; doi:10.3390/antibiotics14121235)
Supplement: Supplementary file 1 [file antibiotics-14-01235-s001.zip › Supplementary File 2.pdf]

Supplementary File 2.

Example of the analytic progression from raw data to codes, subthemes and the logic model

Verbatim Quote →	Initial Code →	Sub-theme/Category Development →	Integration into Logic Model
"The biggest barrier is that we don't have a national policy the current application of which is promoting pharmacists' involvement in AMS"	No AMS policies	Absence of national AMS policies and programs	AMS policy and governance support
"It could be that in our healthcare system, stakeholders are threatened by us [pharmacist] that we will take their position, so they hesitate to include us in AMS."<br><br>"We [pharmacists] should be further involved in One Health initiatives"	Exclusion from initiatives<br><br>Further involvement in one health	Stakeholders' hesitance<br><br>Interdisciplinary collaborations	One Health interdisciplinary collaborations
"Globally in multiple countries what's happening now is that pharmacists have complete dominance in prescription practices and they can generally prescribe for minor conditions."	Prescribing by pharmacists	Prescribing rights and authority	Provision of prescribing rights for minor ailments
"The first basic thing is our own education, our own trainings, we have not had such trainings, neither in our education, nor in our course book we had such things…"	No capacity building	Insufficient trainings, workshops, education	Promotion of AMS trainings, workshops, education
